# Curcumin Ameliorates the Cd-Induced Anxiety-like Behavior in Mice by Regulating Oxidative Stress and Neuro-Inflammatory Proteins in the Prefrontal Cortex Region of the Brain

**DOI:** 10.3390/antiox10111710

**Published:** 2021-10-27

**Authors:** Dhondup Namgyal, Sher Ali, Muhammad Delwar Hussain, Mohsin Kazi, Ajaz Ahmad, Maryam Sarwat

**Affiliations:** 1 Amity Institute of Neuropsychology and Neuroscience, Amity University, Noida 201303, India; dhonamdhonam@gmail.com; 2 Amity Institute of Pharmacy, Amity University, Noida 201303, India; 3 Department of Life Sciences, School of Basic Sciences and Research, Sharda University, Greater Noida 201310, India; sher.ali@sharda.ac.in; 4 Department of Pharmaceutical & Biomedical Sciences, College of Pharmacy, California Health Sciences University, 120 N. Clovis Avenue, Clovis, CA 93612, USA; delwarcc@gmail.com; 5 Department of Pharmaceutics, College of Pharmacy, King Saud University, P.O. Box 2457, Riyadh 11451, Saudi Arabia; mkazi@ksu.edu.sa; 6 Department of Clinical Pharmacy, College of Pharmacy, King Saud University, P.O. Box 2457, Riyadh 11451, Saudi Arabia; aajaz@ksu.edu.sa

**Keywords:** curcumin, cadmium, inflammation, oxidative stress, spatial working memory

## Abstract

Age-related neurodegenerative diseases and vascular dementia are major challenges to the modern health care system. Most neurodegenerative diseases are associated with impaired spatial working memory and anxiety-like behavior. Thus, it is important to understand the underlying cellular mechanisms of neurodegenerative diseases in different regions of the brain to develop an effective therapeutic approach. In our previous research paper, we have reported the ameliorative effect of curcumin in Cd-induced hippocampal neurodegeneration. However, recently many researchers had reported the important role of the prefrontal cortex in higher cognitive functions. Therefore, to look into the cellular mechanism of curcumin protection against Cd-induced prefrontal cortex neurotoxicity, we investigated spatial working memory, anxiety-like behavior and analyzed prefrontal cortex inflammatory markers (IL-6, IL-10, and TNFα), antioxidant enzymes (SOD, GSH, and CAT), and pro-oxidant MDA level. Further, we conducted histological studies of the prefrontal cortex in Swiss albino mice exposed to cadmium (2.5 mg/kg). We observed that curcumin treatment improved the spatial working memory and anxiety-like behavior of mice through reduction of prefrontal cortex neuroinflammation and oxidative stress as well as increasing the number of viable prefrontal cortex neuronal cells. Our result suggests that environmental heavy metal cadmium can induce behavioral impairment in mice through prefrontal cortex cellular inflammation and oxidative stress. We found that curcumin has a potential therapeutic property to mitigate these behavioral and biochemical impairments induced by cadmium.

## 1. Introduction

Neurodegenerative diseases are the major concern of the modern health care system. Though the advancements in the medical field have increased the lifespan of human beings, the occurrence of age-related neurodegenerative diseases and dementia-related illnesses have increased from 4.0% in 1990 to 8.2% in 2019 [1] Depressive disorders (33.8%) and anxiety disorders (19%) are the major contributors to mental illness affecting close to about 197.3 million people (~14.3%) [2]. Several factors including daily light/dark cycle, diet, physical exercise, heavy metal toxicity, and social stress regulate neurodegeneration in both central and peripheral nervous systems, but oxidative stress and neuroinflammations are major causes of neurodegeneration [3,4,5,6,7,8]. In most neurodegenerative disorders, these two processes occur simultaneously and coexist [9,10]. However, the exact pathophysiology of depression and anxiety associated with neurodegenerative disease in patients is poorly understood.

**Figure 1:**
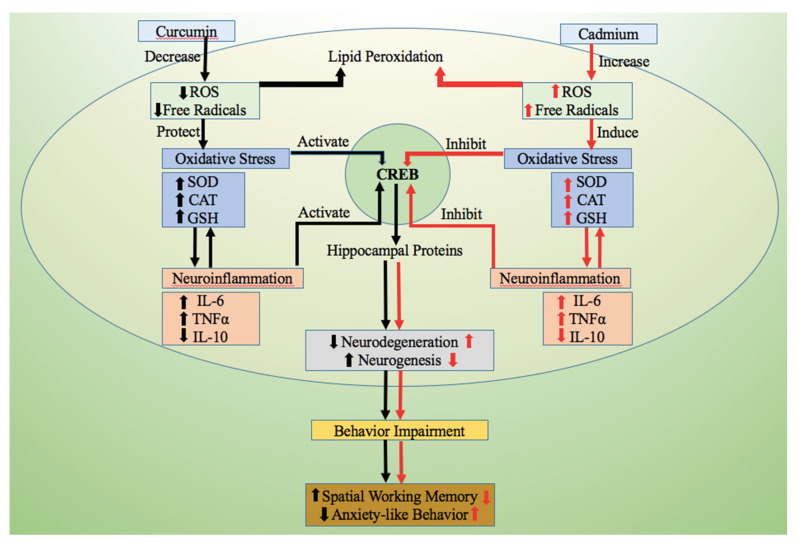
Diagrammatic illustration showing the pathway involved in the Cd-induced behavioral impairment (**right panel**) and the ameliorating effect of curcumin (**left panel**). Upon Cd exposure, the cellular reactive oxygen species (ROS) and free radicals increased, causing oxidative stress, as the cellular antioxidant enzymes (SOD, CAT, and GSH) decreased. Cd exposure also leads to neuroinflammation by increasing the cellular pro-inflammatory markers (IL-6 and TNFα). This causes behavioral impairment and acceleration of neurodegeneration. On curcumin treatment, cellular oxidative stress and neuroinflammation decreased, thereby promoting hippocampal neurogenesis and restoring mice behavior. Reactive oxygen species (ROS), cadmium (Cd), superoxide dismutase (SOD), catalase (CAT), glutathione (GSH), interleukin-6 (IL-6), tumor necrosis factor alpha (TNFα). (Image source BioRender.com; accessed on 28 August 2021).

Earlier, we observed that mice exposed to Cd for 60 days showed impaired locomotor activity and loss of recognition memory, and reduced levels of neurogenesis associated hippocampal proteins [11]. The production of new neurons in the central nervous system (CNS) is regulated by the redox cycle and pro-inflammatory cytokine levels (Figure 1) [12,13]. Several studies have shown that Cd exposure activates the brain microglial cells which in turn induces the production of pro-inflammatory proteins including interleukin-6 (IL-6) and tumor necrosis factor-alpha (TNFα) [14]. Overproduction of these pro-inflammatory proteins leads to neurodegeneration through different cellular signaling pathways [15,16].

The prefrontal cortex regulates and controls a variety of higher cognitive functions including thinking, planning, reasoning, and decision making [17]. Moreover, recent findings suggest that this region of the brain also regulates the formation of short-term spatial working memory and anxiety-like cognitive behavior [18]. However, the effect of Cd on the prefrontal cortex of the brain region has not been studied. Therefore, the main objective of the present study is to investigate the effect of prolonged exposure of Cd on oxidative stress and inflammation in the prefrontal cortex of mice.

Currently, there are many pharmacological interventions for memory improvement and amelioration of anxiety-like behavior associated with neurodegenerative diseases. However, they have drawbacks such as reduced libido, withdrawal symptoms, insomnia, off-target effects, and resistance to the treatment [19]. Therefore, a therapeutic approach with a multi-targeted strategy against neurodegenerative disease-associated behavioral impairment is warranted.

Curcumin is an active component of turmeric (Curcuma longa) which is widely used as a food additive in Indian cuisines and Ayurvedic medicines [20]. Previously, researchers have reported the neuroprotective effect of curcumin in various neurodegenerative diseases [9,21,22,23,24]. In our study on dim light-induced neurodegeneration, we have shown the antioxidant effects of curcumin [25]. Other researchers have also highlighted the anti-inflammatory and antioxidant effects of curcumin [26,27]. Therefore, given the established anti-inflammatory and antioxidant effect of curcumin, the second objective of the present study is to investigate whether curcumin could mitigate the Cd-induced behavioral impairment, neuroinflammation, and oxidative stress in the same experimental animals.

## 2. Materials and Methods

### 2.1. Drugs and Biochemical Reagents

Cadmium chloride and curcumin extract (95%) were purchased from Sisco Research Laboratory (SRL) Pvt. Ltd. (Pune, India). Phosphate buffer saline (PBS), pioglitazone, formaldehyde, 5,5′-dithiobis (2-nitrobenzoic acid) (DTNB), trichloroacetic acid, thiobarbituric acid (TBA), butanol, nitroblue tetrazolium (NBT), disodium hydrogen phosphate, sodium citrate, and hydrogen peroxide (H_2_O_2_) were obtained from Thermo Fisher Scientific (Waltham, MA, USA). Mouse IL-6, IL-10, and TNFα ELISA kits were purchased from Ray Biotech (Peachtree Corners, GA, USA). A polytron tissue homogenizer (Thomas Scientific, Swedesboro, NJ, USA), an ELISA reader (Trans Asia Pvt. Ltd., Mumbai, Maharashtra, India), and a UV spectrometer (Perkin Elmer, Waltham, MA, USA) were used during the experiment.

### 2.2. Experimental Animals

A total of 56 young Swiss Albino mice (25–30 g, 4 weeks of age) were obtained from the animal house of Amity University, Noida. Mice were individually housed in propylene cages (dimensions: 33 × 19 × 14 cm) at an ambient temperature of 22 ± 2 °C and provided with Harlan teklad 8640 food (Madison, WI, USA) and filtered tap water ad libitum. All mice were maintained in a standard light-dark cycle (LD; 12:12 light (150 lux)/dark (0 lux)) during the 60 days of experimentation. All experimental procedures were approved by the Institutional Animal Care and Ethical Committee of Amity University (CPCSEA/IAEC/AIP/2017/03/02). The animals were maintained in accordance with the recommendations of the Committee for the Purpose of Control and Supervision of Experiments on Animals (CPCSEA), India. A total of 56 animals were used, dividing them into 8 groups, each having 7 animals. The group details are given hereunder.

1. Vehicle control = (1% carboxy methyl cellulose)
2. Cd control = (2.5 mg/kg of Cd for 60 days)
3. Cd + Cur20 = (2.5 mg/kg of Cd for 60 days and 20 mg/kg of curcumin for 30 days)
4. Cd + Cur40 = (2.5 mg/kg of Cd for 60 days and 40 mg/kg of curcumin for 30 days)
5. Cd + Cur80 = (2.5 mg/kg of Cd for 60 days and 80 mg/kg of curcumin for 30 days)
6. Cd + Cur160 = (2.5 mg/kg of Cd for 60 days and 160 mg/kg of curcumin for 30 days)
7. Cur160 = (160 mg/kg of curcumin for 30 days)
8. Cd + Pio30 = (2.5 mg/kg of Cd for 60 days and 30 mg/kg of pioglitazone for 30 days)

Cadmium was administered daily from day 1 to 60 and curcumin was administered daily from day 30 to 60.

### 2.3. Behavior Studies

#### 2.3.1. Y-Maze

The y-maze test was employed to analyze the percentage of spontaneous alternation, a measure of the spatial working memory of the animal [28]. The maze makes a Y shape, with three arms of equal lengths and each at an angle of 120° from the other. One of the arms was considered as the start arm and all animals were placed at the end of this arm pointing towards the center of the maze. The animals were subjected to 8 min of testing in the y-maze. The exploration of three different arms in succession was considered as one alternation. Serial arm entries were observed for each animal to calculate the percentage of spontaneous alternations using the following formula.

% spontaneous alternation = total alternations/(total arm entries − 2) × 100

#### 2.3.2. Elevated Plus-Maze

Elevated plus-maze (EPM) is a commonly used apparatus to assess anxiety-like behavior in animals [29]. The EPM apparatus was made up of wood with four arms at 90° to each other. Two open arms and two closed arms of 50 × 10 cm dimensions are enclosed by a 40 cm high wall. After 60 days of the experimental period, all mice were shifted to the pretest arena and kept for 5 min. The mice were then transferred to the EPM placed 50 cm high from the ground. All mice were released in the center of the maze, pointing towards the open arm, and a video recording device was kept above the EPM apparatus to record the number of entries in the arms along with the time spent in each arm for 5 min. Since the open arm is bright and unprotected, whereas the closed arm is shadowy and protected, the mice with high stress remain mostly in the closed arm. Therefore, to investigate the anxiety-like behavior of mice, the % open arm entries and % open arm time was calculated using the following formula.

% open arm entries = number of entries in open arm/(closed arm entries + open arm entries) × 100

% time spent in open arm = time spent in open arm/(time spent in open arm + time spent in closed arm) × 100

### 2.4. Oxidative Stress Test

#### 2.4.1. Lipid Peroxidation

Lipid peroxidation is one of the major causes of neurodegeneration. The extent of lipid peroxidation in the prefrontal region of brain tissue was analyzed quantitatively by employing the method described by Wills [30]. Briefly, the prefrontal brain samples were mixed with 1 mL of trichloroacetic acid (10%) and 1 mL of thiobarbituric acid (0.67%), heated in a boiling water bath for 15 min. Butanol (2:1 *v/v*) was added to the solution. The amount of malondialdehyde (MDA) was measured by the reaction with thiobarbituric (TBA) acid at 532 nm using a UV spectrophotometer. The values were calculated using the molar extinction coefficient of MDA-TBA adduct at 532 nm, which is 155 (mM^−1^cm^−1^).

#### 2.4.2. SOD Activity

Superoxide dismutase (SOD) is an endogenous antioxidant enzyme that catalyzes the superoxide into molecular oxygen (O_2_) and hydrogen peroxide (H_2_O_2_). SOD activity was analyzed by nitroblue tetrazolium (NBT) method which is based on the principle that NBT undergoes a photo-reduction (a blue-colored formazan) by superoxide radicals when exposed to light. It competes with the enzyme SOD for superoxide anions. With the presence of SOD in the reaction mixture, NBT produces a lesser quantity of colored complexes as compared to the control. Prefrontal cortex tissue homogenate (500 μL) was mixed with chloroform (300 μL) and ethanol (500 μL). The mixture was centrifuged at 18,000× *g* for 30 min then 50 μL of supernatant was taken and mixed with 900 μL of SOD reagent (0.1 mmol/L xanthine, 0.1 mmol/L EDTA, 50 mg bovine serum albumin, 25 mmol/L NBT and 40 mmol/L Na_2_CO_3_) (pH 10.2). Further, twenty-five units of xanthine oxidase were added to the mixture and incubated for 20 min at 25 °C. The reaction was stopped by adding 1 mL of CuCl_2_ (0.8 mmol/L) and absorbance was recorded at 560 nm [31].

#### 2.4.3. Catalase Activity

Catalase is another endogenous antioxidant enzyme present in every living organism. This enzyme catalyzes the decomposition of H_2_O_2_ into O_2_ and water (H_2_O). The activity of the catalase enzyme was analyzed by a spectrophotometer at 240 nm. Briefly, 1 mL of the brain homogenate was taken in a test tube and 1.9 mL of the phosphate buffer (50 mM, pH 7.4) was added. The reaction was initiated by the addition of 1 mL of 30 mM H_2_O_2_. The mixture of 2.9 mL of phosphate buffer and 1 mL of H_2_O_2_ without the brain homogenate was taken as blank. The decomposition of H_2_O_2_ resulted in the reduction of absorbance, which was recorded at 240 nm against the blank. The unit of catalase activity was expressed as the amount of enzyme that decomposes 1 μM of H_2_O_2_ per min at 25 °C using the molar coefficient of 43.6 M^−1^ cm^−1^ and the activity was expressed in terms of unit/mg proteins [32].

#### 2.4.4. Glutathione Level

The glutathione (GSH) is an antioxidant enzyme and protects cellular damage from reactive oxygen species (ROS). The total content of GSH in the mice’s prefrontal brain region was analyzed spectrophotometrically at 412 nm. Briefly, in a test tube, the supernatant of the prefrontal brain homogenate and trichloroacetic acid (10% *w*/*v*) were mixed in a 1:1 ratio and centrifuged at 1000× *g* for 10 min at 4 °C. The supernatant (0.5 mL) was mixed with 0.3 M disodium hydrogen phosphate (2 mL) and 0.25 mL of 0.001 M freshly prepared DTNB (5,5′-dithiobis (2-nitrobenzoic acid) dissolved in 1% *w/v* sodium citrate). The absorbance was recorded at 412 nm. A standard curve was plotted using 10–100 μM of the reduced glutathione and the results were expressed as micromoles of reduced glutathione per mg of protein [32].

### 2.5. Analysis of Inflammatory Markers

To investigate the effect of curcumin on Cd-induced inflammation, ELISA-based protein assays for pro-inflammatory markers including TNF-α, IL-6, and IL-10 were carried out (Ray Biotech, Peachtree Corners, GA, USA). These kits are based on the principle of sandwich ELISA. Briefly, the weighted prefrontal tissue was mixed with 300 μL of lysis buffer and homogenized for 30 s and centrifuged at 16,000 rpm for 20 min at 4 °C. All samples were assayed in triplicate. Absorbance was measured at 450 nm with an ELISA plate reader (Trans Asia Pvt. Ltd., Mumbai, Maharashtra, India). The concentrations for IL-6 and IL-10 were expressed as pg mL^−1^ and that of TNF-α was presented as ng mL^−1^.

### 2.6. Morphometric and Histopathological Analyses

The mice from each group were randomly selected and sacrificed in the morning time (09:00–11:00 a.m.) to avoid the interference of stress hormones. For histological analysis, dissected mice brains were fixed in methanol/chloroform/acetic acid solution (6:3:1) and stored in 10% formaldehyde. Afterward, the brain tissues were dehydrated in ethanol and clarified using xylene. The brain tissues were embedded in Paraplast Plus and coronal sections of 3-m thickness were cut with a microtome, slides were processed and stained with hematoxylin (H) and eosin (E). After dehydration using ethanol series, slides were mounted for microscopic examination. Morphometry was carried out by analyzing the prefrontal region of the brain. From each brain sample, five sections were stained for analysis. The neurotoxic effect of Cd and the neuroprotective effect of curcumin on neuronal cells were qualitatively analyzed using an Olympus BX43 light microscope (Olympus, Tokyo, Japan). The presence of hypereosinophilic cytoplasm or pyknotic nuclei was used to identify non-viable neurons.

### 2.7. Statistical Analysis

Values are represented as mean ± standard deviation (*n* = 7). A non-parametric one-way ANOVA test was employed to compare the behavior results between control and various treatment groups. One-way ANOVA with post-hoc Dunnett’s test was employed to compare the prefrontal inflammatory markers and antioxidant enzymes between control and various treatment groups. The data were analyzed using Graphpad Prism-8 software. The values of *p* < 0.05 represent a statistically significant difference between the groups.

## 3. Results

### 3.1. Curcumin Increases the Body Weight in Cd Exposed Mice

Fluctuation in body weight is one of the characteristics of patients with neurodegenerative diseases. When we investigated the percentage of body weight gained during the experiment period (60 days), we observed a significant reduction in the Cd-treated mice as compared to the vehicle control group (F_7,48_ = 21.80, *p* < 0.001). Hence, showing a negative impact of long-term Cd exposure on animal metabolism.

**Figure 2:**
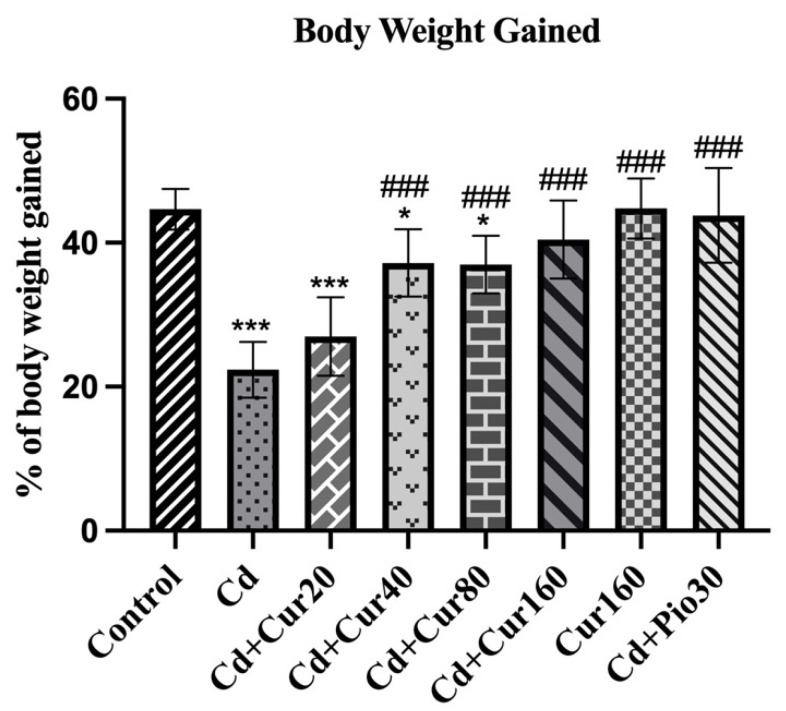
Effect of curcumin on Cd exposed mice exhibiting % body weight gained during the experiment. Each column represents the mean ± SEM of 7 animals. One-way ANOVA followed by Dunnett’s post-test; * *p* < 0.05 and *** *p* < 0.001 showing significant differences between control and treatment group. ^###^ *p* < 0.001 showing significant differences between Cd exposed group and curcumin treated groups.

Treatment with different concentrations of curcumin had caused restoration of body weight of the experimental animals (F_7,48_ = 21.80, *p* < 0.001) as the mean percentage of body weight gained was significantly increased in the curcumin treated group as compared to the Cd exposed mice. The percentage of body weight gained in the Cd + Cur160 group of mice is almost similar to the vehicle control group (Figure 2). Thus, exhibiting the potential therapeutic effect of curcumin in regulating body weight and metabolism.

### 3.2. Curcumin Improves Spatial Working Memory of Mice Exposed to Cd

**Figure 3:**
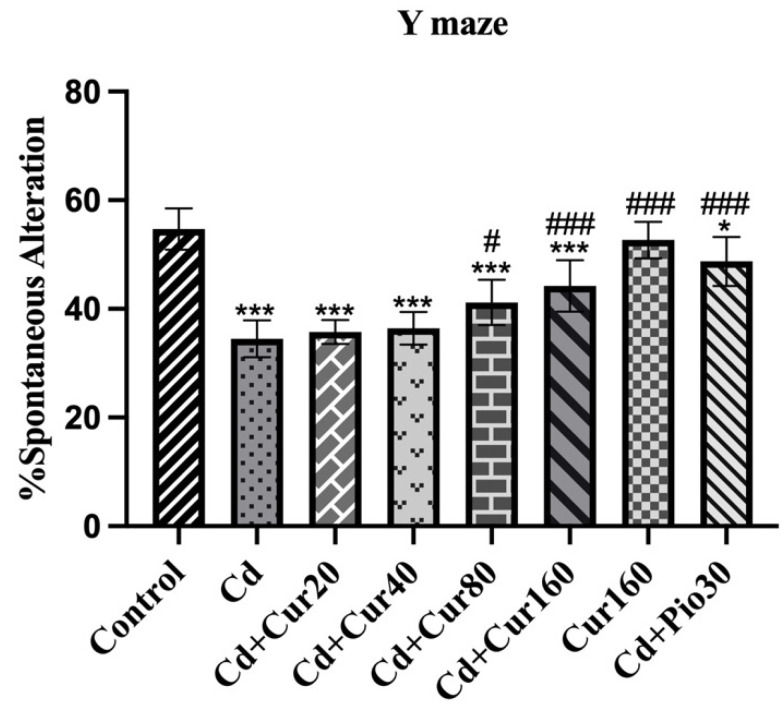
Effect of curcumin on Cd exposed mice depicting spatial working memory of rodents. Each column represents the mean ± SEM of 7 animals. One-way ANOVA followed by Dunnett’s post-test; * *p* < 0.05 and *** *p* < 0.001 showing significant differences between control and treatment group. ^#^ *p* < 0.05 and ^###^ *p* < 0.001 and showing significant differences between Cd exposed group and curcumin treated groups.

In most neurodegenerative disease patients, there is a severe deterioration in the behaviors, including impaired memory and learning ability. Through the y-maze test, we see a significant reduction in the percentage of spontaneous alternation in the Cd-treated mice as compared to normal vehicle control groups (F_4,48_ = 26.74, *p* < 0.001). Thus, Cd-treated mice show impaired spatial working memory. However, treatment with different concentrations of curcumin had significantly increased the percentage of spontaneous alternation in the Cd-treated mice as compared to normal vehicle control groups and curcumin-treated group (F_4,48_ = 26.74, *p* < 0.001) (Figure 3). Therefore, this finding indicates that curcumin can mitigate the Cd-induced spatial memory impairment.

### 3.3. Curcumin Ameliorates Cd-Induced Anxiety-like Behavior

**Figure 4:**
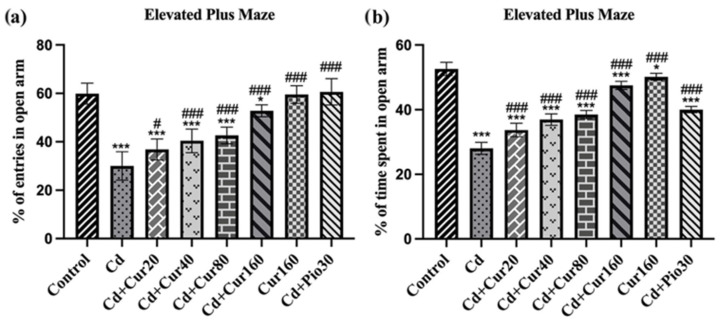
Effect of curcumin on Cd exposed mice showing anxiety-like behavior; (**a**) % of entry in open arm and (**b**) % of the time in open arm. Each column represents the mean ± SEM of 7 animals. One-way ANOVA followed by Dunnett’s post-test; * *p* < 0.05 and *** *p* < 0.001 showing significant differences between control and treatment group. ^#^ *p* < 0.05 and ^###^ *p* < 0.001 and showing significant differences between Cd ex-posed group and curcumin treated groups.

Anxiety-like behavior is a common syndrome of patients with neurodegenerative disease and cognitive impairment. The result of the EPM test revealed that the percentage of open arm entries (F_7,48_ = 50.61, *p* < 0.001) and duration in the open arm (F_7,48_ = 197.10, *p* < 0.001) are significantly lower in the Cd treated mice as compared to the normal vehicle control group. This indicated that prolonged Cd exposure has deleterious effects on the behavior of animals and causes increased anxiety-like behavior. However, treatment with different curcumin concentrations had effectively protected the Cd-induced anxiety-like behavior as the percentage of open arm entry (Figure 4a) and duration (Figure 4b) are significantly increased in the curcumin-treated groups (*p* < 0.001). Therefore, the result of EPM depicts the potential of curcumin in regulating the behavior of Cd exposed animals.

### 3.4. Curcumin Protects Cd-Induced Prefrontal Cortex Oxidative Stress

**Figure 5:**
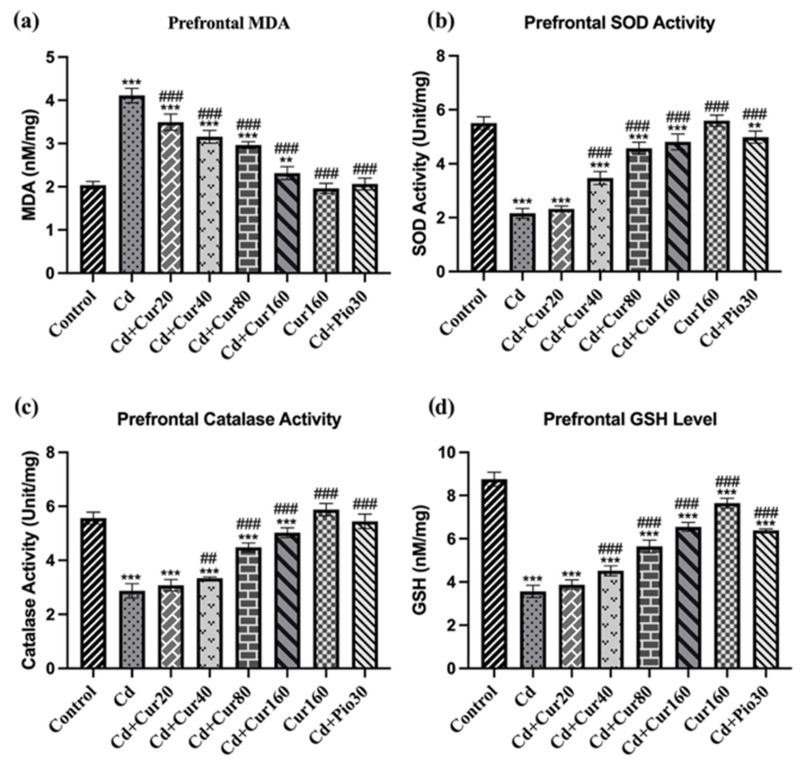
Effect of curcumin on Cd exposed mice showing oxidative stress; (**a**) MDA (**b**) SOD (**c**) CAT and (**d**) GSH. Each column represents the mean ± SEM of 7 animals. One-way ANOVA followed by Dunnett’s post-test; * *p* < 0.05, ** *p* < 0.01 and *** *p* < 0.001 showing significant differences between control and treatment group. ^##^ *p* < 0.01 and ^###^ *p* < 0.001 and showing significant differences between Cd ex-posed group and curcumin treated groups.

The prefrontal cortex antioxidant enzyme analyses revealed that mice exposed to Cd for 60 days had significantly increased pro-oxidant MDA levels as compared to normal vehicle control groups (F_7,40_ = 196.20, *p* < 0.001) (Figure 5a). Moreover, Cd exposure had also significantly reduced the prefrontal cortex antioxidant enzymes, including SOD (F_7,40_ = 230.70, *p* < 0.001) (Figure 5b), CAT (F_7,40_ = 200.00, *p* < 0.001) (Figure 5c), and GSH (F_7,40_ = 338.90, *p* < 0.001) (Figure 5d) as compared to the normal control group. These results exhibit that prolonged Cd exposure leads to neurodegeneration in the prefrontal cortex due to the build-up of oxidative stress. However, treatment of different concentrations of curcumin had significantly reduced the pro-oxidant MDA (*p* < 0.001) level and increased the activity of the antioxidant enzymes including SOD, CAT, and GSH (*p* < 0.001) in the prefrontal cortex brain sample as compared to Cd exposed mice. Thus, oral administration of curcumin mitigates the Cd-induced oxidative stress.

### 3.5. Curcumin Protects Cd-Induced Prefrontal Cortex Neuroinflammation

**Figure 6:**
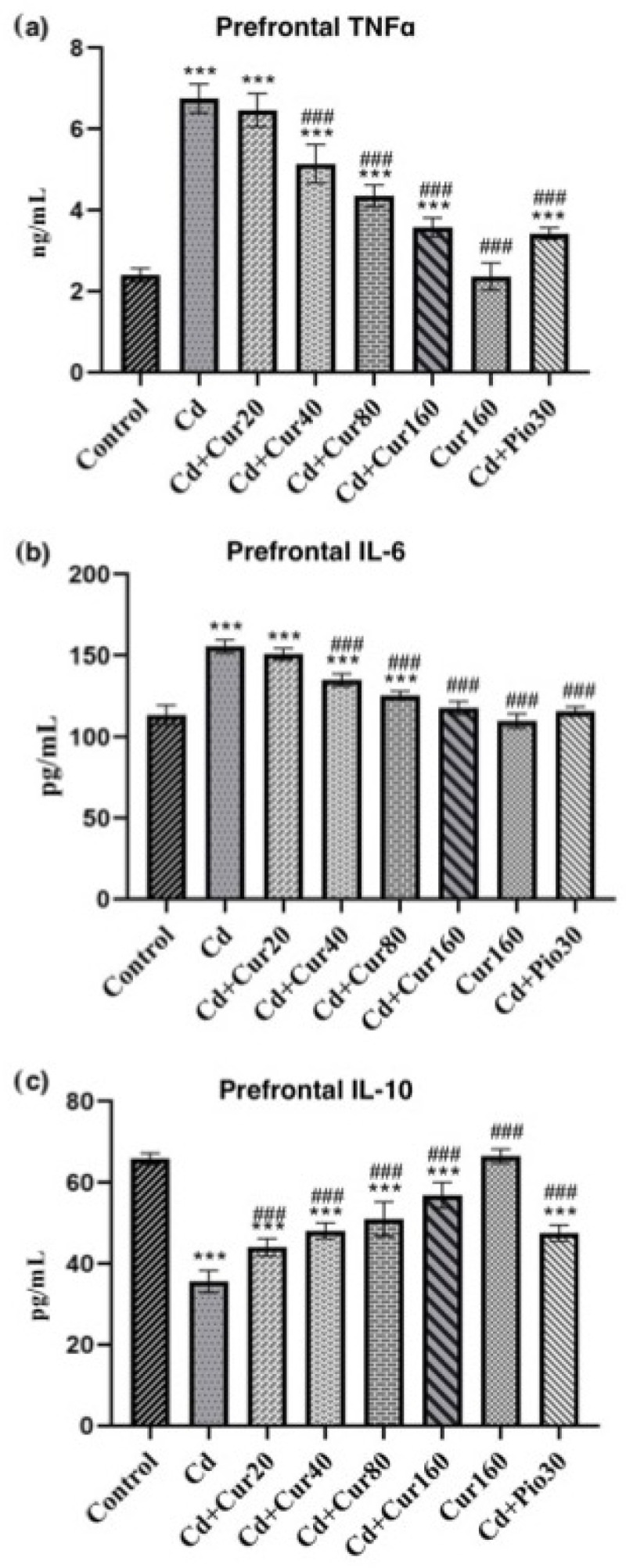
Effect of curcumin on Cd exposed mice showing the status of inflammatory markers; (**a**) IL-6 (**b**) TNFα (**c**) IL-10. Each column represents the mean ± SEM of 7 animals. One-way ANOVA followed by Dunnett’s post-test; *** *p* < 0.001 showing significant differences between control and treatment group. ^###^ *p* < 0.001 and showing significant differences between Cd exposed group and curcumin treated groups.

In most neurodegenerative diseases, the neuroinflammation process is accompanied by oxidative stress and thereby causes behavioral impairment in patients. Cd exposure for 60 days had significantly increased the prefrontal cortex pro-inflammatory markers IL-6 (F_7,40_ = 252.70, *p* < 0.001) (Figure 6a) and TNFα (F_7,40_ = 147.50, *p* < 0.001) (Figure 6b) as compared to the normal vehicle control group. Moreover, the anti-inflammatory cytokine IL-10 (F_7,40_ = 358.70, *p* < 0.001) (Figure 6c) was reduced in the prefrontal region of the Cd-treated mice as compared to the normal control groups. These findings revealed that exposure to Cd for a long time could lead to inflammation-induced neurodegeneration through reduction of anti-inflammatory IL-10 and elevation of pro-inflammatory markers (IL-6 and TNFα). However, treatment of different concentrations of curcumin had significantly increased the IL-10 (*p* < 0.001) and reduced the IL-6 and TNFα (*p* < 0.001) as compared to Cd exposed mice. Therefore, these results suggest that curcumin controls neuroinflammation in the Cd-exposed mice using different pathways.

### 3.6. Curcumin Protects the Neurodegeneration Induced by Cd

**Figure 7:**
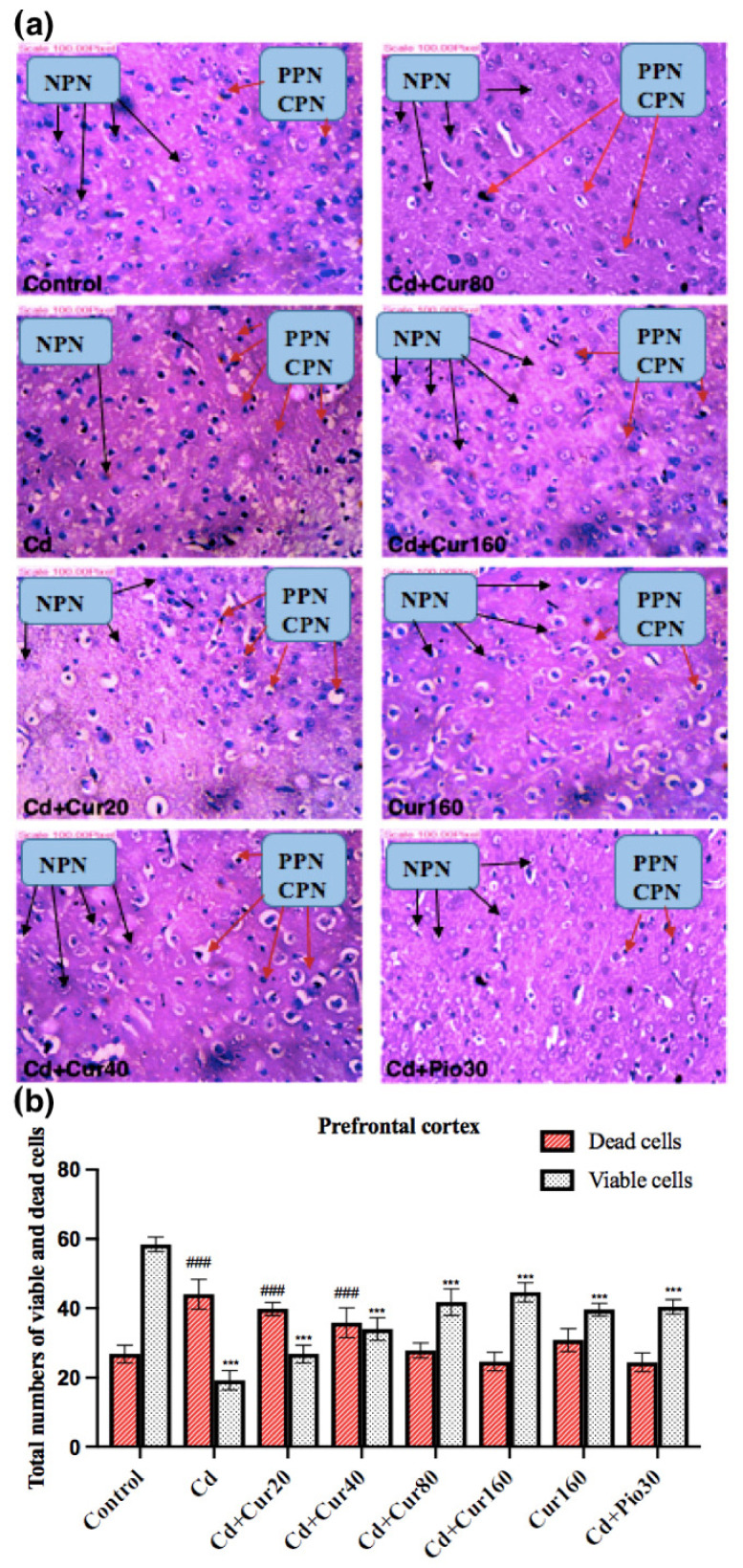
Effect of curcumin on cadmium exposed animals’ prefrontal cortex brain region. (**a**) In the Cd exposed groups, more pyknotic prefrontal neurons (PPN) and chromatolysis prefrontal neurons (CPN) were seen in the prefrontal cortex region (red arrows). Curcumin treatment had significantly increased the normal prefrontal neurons (NPN) (black arrows). (**b**) The total number of viable and dead neurons in the prefrontal cortex region of different groups of mice. *** *p* < 0.001 showing significant differences of viable cells between control and treatment group. ^###^ *p* < 0.001 and showing significant differences of dead cells between Cd exposed group and curcumin treated groups.

If there is oxidative stress and neuroinflammation, it will most likely induce neurodegeneration. Employing H and E staining, we detected severe degeneration of prefrontal cortex neurons in mice exposed to Cd for 60 days. The number of neurons showing characteristics of chromatolysis and pyknosis was more in the prefrontal cortex of mice exposed to the Cd as compared to the normal control group (Figure 7a). The total number of dead cells was higher in the Cd exposed group as compared to the control and curcumin-treated group (Figure 7b). In addition, the treatment of curcumin effectively prevented the morphological disruption of neuronal cells in the prefrontal cortex of Cd-exposed mice (Figure 7a). This is because we noticed a reduced number of dead cells and an increased number of viable cells in the curcumin-treated groups (Figure 7b). Thus, prolonged exposure of mice to Cd had a deleterious effect on the structural and functional development of the prefrontal cortex neurons. Since the prefrontal cortex of the brain plays a vital role in higher cognitive functions, the impairment of cognitive and non-cognitive behaviors of mice in the Cd-treated group might be mediated through neuronal degeneration (ND) in this region of the brain. Thus, ND is the result of the Cd and neuro-protection is the outcome of curcumin.

## 4. Discussion

As mentioned earlier, Cd causes ND but the same is ameliorated by curcumin. This is evident from the prolonged exposure of the mice to different doses of Cd that resulted in decreased weight gain, impaired spatial working memory, and increased anxiety-like behavior, and increased prefrontal cortex oxidative stress and neuroinflammation. Moreover, exposure to Cd had distorted the morphological structure of prefrontal neurons, as the total number of dead cells was found to be increased in the Cd-exposed mice. The neuroinflammation was evident from the behavioral impairments induced by Cd as evidenced by the y-maze and elevated plus-maze test. The ameliorative effect of curcumin was evidenced because treatment of curcumin reduces the prefrontal cortex oxidative stress and neuroinflammation. This was supported by an increased endogenous antioxidant enzyme (SOD, catalase, and GSH) and reduced pro-inflammatory markers including IL-6 and TNFα in Cd exposed mice. Moreover, the treatment of different concentrations of curcumin had increased the total number of viable neuronal cells in the prefrontal cortex of mice. Earlier, we reported that exposure to Cd (2.5 mg/kg) for 60 days had impaired the recognition memory and locomotor activity of mice which is substantiated by reduction of hippocampal neurogenesis [11]. Therefore, the Cd-induced behavioral impairment seems to be multifactorial, involving prefrontal cortex oxidative stress, neuroinflammation, and reduction of hippocampal neurogenesis. Other workers have also reported that Cd impaired cognitive behavior of mice is corroborated with the reduction of hippocampal neurogenesis [33,34]. During the experiment, the percentage of body weight gained suggests that Cd exposure causes weight loss in the experimental animals. Since the weight gain and loss are dependent on the rate of metabolism, prolonged exposure to Cd might have disrupted the metabolism of mice. In accordance with our study, researchers have reported that exposure to Cd causes bodyweight reduction in birds [35] and rats [36]. These observations were in accordance with the results of decreased protein synthesis and growth after exposure to cadmium [37]. We noticed different concentrations of curcumin are effective for the restoration of weight loss in the Cd exposed mice. Several researchers have reported that curcumin administration protects the weight loss in other mouse models [38,39].

The y-maze test is commonly used to investigate spontaneous alternation percentage, a measure of spatial working memory of animals. We found that the ability to form a spatial working memory is lower in the Cd exposed mice. Earlier, researchers have reported that Cd exposure causes impairment in the memory and learning ability in experimental animals [20,40]. Moreover, the result of the elevated plus-maze test also revealed that mice exposed to Cd had higher anxiety-like behavior, as they spent more time in closed arms as compared to normal control groups [41,42]. With the treatment of different concentrations of curcumin, we observed increased spontaneous alternation percentage (spatial working memory) and reduced anxiety-like behavior in mice. The curcumin treatment has been reported to improve both cognitive and non-cognitive behavior of animals [11,43].

Oxidative stress is one of the major causes of neurodegeneration and behavioral impairment as mentioned earlier [4,44]. In our body, the antioxidant enzymes ensure the regulation of the pro-oxidants (reactive oxygen species (ROS) and free radicals). At times, the production of these enzymes is not able to overcome the number of pro-oxidants produced, leading to oxidative stress-induced neuronal degeneration. In our current study, we observed that Cd exposure increased the oxidative stress in the prefrontal region of the brain through the reduction of endogenous antioxidant enzyme activity (SOD, CAT, and GSH). Moreover, Cd had significantly increased the pro-oxidant MDA level as compared to the normal control and curcumin-treated groups. This result affirms that prolonged exposure to environmental Cd can induce neurodegeneration and behavioral impairment through prefrontal cortex oxidative stress. Our previous study reported an increased hippocampal oxidative stress in mice after Cd exposure [11]. However, treatment of different concentrations of curcumin had effectively protected the Cd-induced oxidative stress supporting earlier studies on different animal models [45,46,47].

Oxidative stress is accompanied by neuroinflammation and this is another major factor of neurodegeneration [48,49]. We found that prolonged exposure to Cd had significantly increased the prefrontal cortex pro-inflammatory markers (IL-6 and TNFα) and reduced the anti-inflammatory cytokine IL-10. This shows that the behavior impairment in the Cd exposed mice was mediated through oxidative stress and neuroinflammation in the prefrontal region of the brain. Previously, researchers have reported similar findings where Cd exposure leads to inflammation and behavior impairment in experimental animals [50]. In addition, our current study revealed that treatment with different curcumin concentrations had significantly reduced the pro-inflammatory markers and increased the anti-inflammatory cytokines in the Cd-exposed mice. Other studies have also revealed the anti-inflammatory property of curcumin in different animal models [51,52,53].

We also noticed that Cd exposure had increased the number of degenerated neurons in the prefrontal cortex region of the brain through oxidative stress and neuroinflammation. Moreover, other studies had also reported Cd-induced morphological disruption of neuronal cells in the prefrontal cortex of rodents [54]. However, treatment of different curcumin concentrations had significantly restored the morphological damage induced by the Cd as it is evident that the total number of viable cells was increased in the curcumin-treated group as compared to the Cd exposed group. These findings are in accordance with our previous report where we showed that treatment of different curcumin concentrations had increased the % viable cells in the hippocampal region of the brain in Cd [11] and dim light at night exposed mice [25]. Thus, curcumin plays an effective role as a natural therapeutic drug to combat Cd-induced behavioral impairment by regulating oxidative stress and modulating inflammatory markers.

## 5. Conclusions

Based on the present data and several earlier studies, it is evidenced that exposure to heavy metal Cd could lead to the impairment of both cognitive and non-cognitive behavior of mice through oxidative stress and neuroinflammation in the prefrontal cortex and reduced hippocampal neurogenesis. Curcumin, on the other hand, was found to improve the behavior of mice through reduction of prefrontal cortex oxidative stress and neuroinflammation as well as promotion of hippocampal neurogenesis. Therefore, curcumin supplementation in food and diet could reduce the deleterious effect of heavy metal exposure. This, however, calls for a careful calibration of the dose of curcumin if given in the context of overall exposure to environmental heavy metals.

## Data Availability

Data contained within the article.

## References

[1] Singh G., Sharma M., Kumar G.A., Rao N.G., Prasad K., Mathur P., Dandona L. (2021). The burden of neurological disorders across the states of India: The Global Burden of Disease Study 1990–2019. Lancet Glob. Health.

[2] Sagar R., Dandona R., Gururaj G., Dhaliwal R.S., Singh A., Ferrari A., Dua T., Ganguli A., Varghese M., Chakma J.K. (2020). The burden of mental disorders across the states of India: The global burden of disease study 1990–2017. Lancet Psychiatry.

[3] Gelders G., Baekelandt V., Van der Perren A. (2018). Linking neuroinflammation and neurodegeneration in Parkinson’s disease. J. Immunol. Res..

[4] Yaribeygi H., Panahi Y., Javadi B., Sahebkar A. (2018). The underlying role of oxidative stress in neurodegeneration: A mechanistic review. CNS Neurol. Disord. Drug Targets.

[5] Amraie E., Pouraboli I., Rajaei Z. (2020). Neuroprotective effects of Levisticum officinale on LPS-induced spatial learning and memory impairments through neurotrophic, anti-inflammatory, and antioxidant properties. Food Funct..

[6] Uddin M.S., Al Mamun A., Kabir M.T., Ahmad J., Jeandet P., Sarwar M.S., Ashraf G.M., Aleya L. (2020). Neuroprotective role of polyphenols against oxidative stress-mediated neurodegeneration. Eur. J. Pharmacol..

[7] Namgyal D., Sarwat M., Sarwat M., Sumaiya S. (2020). Saffron as a neuroprotective agent. Saffron: The Age Old Panacea in New Light.

[8] Fakhri S., Piri S., Moradi S.Z., Khan H. (2021). Phytochemicals targeting oxidative stress, interconnected neuroinflammatory and neuroapoptotic pathways following radiation. Curr. Neuropharmacol..

[9] Yao Y., Chinnici C., Tang H., Trojanowski J.Q., Lee V.M., Praticò D. (2004). Brain inflammation and oxidative stress in a transgenic mouse model of Alzheimer-like brain amyloidosis. J. Neuroinflamm..

[10] Behl T., Makkar R., Sehgal A., Singh S., Sharma N., Zengin G., Bungau S., Andronie-Cioara F.L., Munteanu M.A., Brisc M.C. (2021). Current trends in neurodegeneration: Cross talks between oxidative stress, cell death, and inflammation. Int. J. Mol. Sci..

[11] Namgyal D., Ali S., Mehta R., Sarwat M. (2020). The neuroprotective effect of curcumin against Cd-induced neurotoxicity and hippocampal neurogenesis promotion through CREB-BDNF signaling pathway. Toxicology.

[12] Shetty A.K., Attaluri S., Kodali M., Shuai B., Shetty G.A., Upadhya D., Hattiangady B., Madhu L.N., Upadhya R., Bates A. (2020). Monosodium luminol reinstates redox homeostasis, improves cognition, mood and neurogenesis, and alleviates neuro-and systemic inflammation in a model of Gulf War Illness. Redox Biol..

[13] Sharma N., Biswas S., Al-Dayan N., Alhegaili A.S., Sarwat M. (2021). Antioxidant role of kaempferol in prevention of hepatocellular carcinoma. Antioxidants.

[14] Khan A., Ikram M., Muhammad T., Park J., Kim M.O. (2019). Caffeine modulates cadmium-induced oxidative stress, neuroinflammation, and cognitive impairments by regulating Nrf-2/HO-1 in vivo and in vitro. J. Clin. Med..

[15] Taylor J.M., Main B.S., Crack P.J. (2013). Neuroinflammation and oxidative stress: Co-conspirators in the pathology of Parkinson’s disease. Neurochem. Int..

[16] Spagnuolo C., Moccia S., Russo G.L. (2018). Anti-inflammatory effects of flavonoids in neurodegenerative disorders. Eur. J. Med. Chem..

[17] Liu W.Z., Zhang W.H., Zheng Z.H., Zou J.X., Liu X.X., Huang S.H., You W.J., He Y., Zhang J.Y., Wang X.D. (2020). Identification of a prefrontal cortex-to-amygdala pathway for chronic stress-induced anxiety. Nat. Commun..

[18] Funahashi S. (2017). Working memory in the prefrontal cortex. Brain Sci..

[19] Penn E., Tracy D.K. (2012). The drugs don’t work? Antidepressants and the current and future pharmacological management of depression. Ther. Adv. Psychopharmacol..

[20] Hewlings S.J., Kalman D.S. (2017). Curcumin: A review of its effects on human health. Foods.

[21] Sundaram J.R., Poore C.P., Sulaimee N.H., Pareek T., Cheong W.F., Wenk M.R., Pant H.C., Frautschy S.A., Low C.M., Kesavapany S. (2017). Curcumin ameliorates neuroinflammation, neurodegeneration, and memory deficits in p25 transgenic mouse model that bears hallmarks of Alzheimer’s disease. J. Alzheimer’s Dis..

[22] Abrahams S., Haylett W.L., Johnson G., Carr J.A., Bardien S. (2019). Antioxidant effects of curcumin in models of neurodegeneration, aging, oxidative and nitrosative stress: A review. Neuroscience.

[23] Namgyal D., Chandan K., Ali S., Mehta R., Sarwat M. (2020). Curcumin improves the behavior and memory in mice by modulating the core circadian genes and their associated micro-RNAs. J. Pharmacol. Pharmacother..

[24] Namgyal D., Chandan K., Ali S., Ahmad A., Hashim M.J., Sarwat M. (2021). Aberrant Lighting Causes Anxiety-like Behavior in Mice but Curcumin Ameliorates the Symptoms. Animals.

[25] Namgyal D., Chandan K., Sultan A., Aftab M., Ali S., Mehta R., El-Serehy H.A., Al-Misned F.A., Sarwat M. (2020). Dim light at night induced neurodegeneration and ameliorative effect of curcumin. Cells.

[26] Santana-Martínez R.A., Silva-Islas C.A., Fernández-Orihuela Y.Y., Barrera-Oviedo D., Pedraza-Chaverri J., Hernández-Pando R., Maldonado P.D. (2019). The therapeutic effect of curcumin in quinolinic acid-induced neurotoxicity in rats is associated with BDNF, ERK1/2, Nrf2, and antioxidant enzymes. Antioxidants.

[27] Voulgaropoulou S.D., van Amelsvoort T.A., Prickaerts J., Vingerhoets C. (2019). The effect of curcumin on cognition in Alzheimer’s disease and healthy aging: A systematic review of pre-clinical and clinical studies. Brain Res..

[28] Prieur E.A., Jadavji N.M. (2019). Assessing spatial working memory using the spontaneous alternation Y-maze test in aged male mice. Bio Protoc..

[29] Carola V., D’Olimpio F., Brunamonti E., Mangia F., Renzi P. (2002). Evaluation of the elevated plus-maze and open-field tests for the assessment of anxiety-related behaviour in inbred mice. Behav. Brain Res..

[30] Wills E.D. (1966). Mechanisms of lipid peroxide formation in animal tissues. Biochem. J..

[31] Weydert C.J., Cullen J.J. (2010). Measurement of superoxide dismutase, catalase and glutathione peroxidase in cultured cells and tissue. Nat. Protoc..

[32] Luis A., Corpas F.J., López-Huertas E., Palma J.M., Gupta D.K., Palma J.M., Corpas F.J. (2018). Plant superoxide dismutases: Function under abiotic stress conditions. Antioxidants and Antioxidant Enzymes in Higher Plants.

[33] Wang H., Abel G.M., Storm D.R., Xia Z. (2019). Cadmium exposure impairs adult hippocampal neurogenesis. Toxicol. Sci..

[34] Wang H., Matsushita M.T., Zhang L., Abel G.M., Mommer B.C., Huddy T.F., Storm D.R., Xia Z. (2020). Inducible and conditional stimulation of adult hippocampal neurogenesis rescues cadmium-induced impairments of adult hippocampal neurogenesis and hippocampus-dependent memory in mice. Toxicol. Sci..

[35] Sant’Ana M.G., Moraes R., Bernardi M.M. (2005). Toxicity of cadmium in Japanese quail: Evaluation of body weight, hepatic and renal function, and cellular immune response. Environ. Res..

[36] Yu W., Xum Z., Gaomm Q., Xu Y., Wang B., Dai Y. (2020). Protective role of wogonin against cadmium induced testicular toxicity: Involvement of antioxidant, anti-inflammatory and anti-apoptotic pathways. Life Sci..

[37] El-Demerdash F.M., Yousef M.I., Kedwany F.S., Baghdadi H.H. (2004). Cadmium-induced changes in lipid peroxidation, blood hematology, biochemical parameters and semen quality of male rats: Protective role of vitamin E and β-carotene. Food Chem. Toxicol..

[38] Sugimoto K., Hanai H., Tozawa K., Aoshi T., Uchijima M., Nagata T., Koide Y. (2002). Curcumin prevents and ameliorates trinitrobenzene sulfonic acid–induced colitis in mice. Gastroenterology.

[39] Billerey-Larmonier C., Uno J.K., Larmonier N., Midura A.J., Timmermann B., Ghishan F.K., Kiela P.R. (2008). Protective effects of dietary curcumin in mouse model of chemically induced colitis are strain dependent. Inflamm. Bowel Dis..

[40] Wang H., Zhang L., Abel G.M., Storm D.R., Xia Z. (2018). Cadmium exposure impairs cognition and olfactory memory in male C57BL/6 mice. Toxicol. Sci..

[41] Zhang T., Gao X., Luo X., Li L., Ma M., Zhu Y., Zhao L., Li R. (2019). The effects of long-term exposure to low doses of cadmium on the health of the next generation of mice. Chem. Biol. Interact..

[42] Lamtai M., Azirar S., Zghari O., Ouakki S., El Hessni A., Mesfioui A., Ouichou A. (2021). Melatonin ameliorates cadmium-induced affective and cognitive impairments and hippocampal oxidative stress in rat. Biol. Trace Elem. Res..

[43] Shen J.D., Wei Y., Li Y.J., Qiao J.Y., Li Y.C. (2017). Curcumin reverses the depressive-like behavior and insulin resistance induced by chronic mild stress. Metab. Brain Dis..

[44] Solanki N., Salvi A., Patki G., Salim S. (2017). Modulating oxidative stress relieves stress-induced behavioral and cognitive impairments in rats. Int. J. Neuropsychopharmacol..

[45] Wang Y.L., Ju B., Zhang Y.Z., Yin H.L., Liu Y.J., Wang S.S., Zeng Z.L., Yang X.P., Wang H.T., Li J.F. (2017). Protective effect of curcumin against oxidative stress-induced injury in rats with parkinson’s disease through the Wnt/β-catenin signaling pathway. Cell. Physiol. Biochem..

[46] Ikram M., Saeed K., Khan A., Muhammad T., Khan M.S., Jo M.G., Rehman S.U., Kim M.O. (2019). Natural dietary supplementation of curcumin protects mice brains against ethanol-induced oxidative stress-mediated neurodegeneration and memory impairment via Nrf2/TLR4/RAGE signaling. Nutrients.

[47] Damiano S., Longobardim C., Andretta E., Prisco F., Piegari G., Squillacioti C., Montagnaro S., Pagnini F., Badino P., Florio S. (2021). Antioxidative effects of curcumin on the hepatotoxicity induced by Ochratoxin A in rats. Antioxidants.

[48] McManus R.M., Heneka M.T. (2017). Role of neuroinflammation in neurodegeneration: New insights. Alzheimer’s Res. Ther..

[49] Czarny P., Wigner P., Galecki P., Sliwinski T. (2018). The interplay between inflammation, oxidative stress, DNA damage, DNA repair and mitochondrial dysfunction in depression. Prog. Neuropsychopharmacol. Biol. Psychiatry.

[50] Hao R., Song X., Li F., Tan X., Sun-Waterhouse D., Li D. (2020). Caffeic acid phenethyl ester reversed cadmium-induced cell death in hippocampus and cortex and subsequent cognitive disorders in mice: Involvements of AMPK/SIRT1 pathway and amyloid-tau-neuroinflammation axis. Food Chem. Toxicol..

[51] Yang H., Du Z., Wang W., Song M., Sanidad K., Sukamtoh E., Zheng J., Tian L., Xiao H., Liu Z. (2017). Structure–activity relationship of curcumin: Role of the methoxy group in anti-inflammatory and anticolitis effects of curcumin. J. Agric. Food Chem..

[52] Maiti P., Paladugu L., Dunbar G.L. (2018). Solid lipid curcumin particles provide greater anti-amyloid, anti-inflammatory and neuroprotective effects than curcumin in the 5xFAD mouse model of Alzheimer’s disease. BMC Neurosci..

[53] Gao Y., Zhuang Z., Lu Y., Tao T., Zhou Y., Liu G., Wang H., Zhang D., Wu L., Dai H. (2019). Curcumin mitigates neuro-inflammation by modulating microglia polarization through inhibiting TLR4 axis signaling pathway following experimental subarachnoid hemorrhage. Front. Neurosci..

[54] Afifi O.K., Embaby A.S. (2016). Histological study on the protective role of ascorbic acid on cadmium induced cerebral cortical neurotoxicity in adult male albino rats. J. Microsc. Ultrastruct..

